# Comparison of intranasal dexmedetomidine versus oral midazolam for premedication in pediatric patients: an updated meta-analysis with trial-sequential analysis

**DOI:** 10.1016/j.bjane.2024.844520

**Published:** 2024-05-25

**Authors:** Eduardo Maia Martins Pereira, Tatiana Souza do Nascimento, Mariana Gaya da Costa, Eric Slawka, Carlos Galhardo Júnior

**Affiliations:** a Universidade Federal de Minas Gerais, Departamento de Medicina, Belo Horizonte, MG, Brazil; b Hospital Federal Cardoso Fontes, Departamento de Anestesiologia, Rio de Janeiro, RJ, Brazil; c University Medical Center of Groningen, Department of Anesthesiology, Groningen, The Netherlands; d Universidade Federal do Rio de Janeiro, Departamento de Medicina, Rio de Janeiro, RJ, Brazil; e McMaster University & DeGroote Schol of Medicine, Department of Anesthesiology, Hamilton, Canada

**Keywords:** Dexmedetomidine, Meta-analysis, Midazolam, Pediatrics, Premedication

## Abstract

**Background:**

Midazolam is routinely used as preanesthetic medication in pediatric patients. Recently, dexmedetomidine has emerged as an alternative as a premedicant. We aimed to add more evidence about the efficacy and safety of two common routes of administration for pediatric premedication: oral midazolam versus intranasal dexmedetomidine.

**Methods:**

We systematically searched Randomized Controlled Trials (RCTs) involving patients ≤ 18 years old undergoing preanesthetic medication and comparing intranasal dexmedetomidine with oral midazolam. Risk Ratio (RR) and Mean Difference (MD) with 95% Confidence Intervals (95% CI) were computed using a random effects model. Trial-sequential analyses were performed to assess inconsistency.

**Results:**

Sixteen RCTs (1,239 patients) were included. Mean age was 5.5 years old, and most procedures were elective. There was no difference in satisfactory induction or mask acceptance (RR = 1.15, 95% CI 0.97–1.37; *p* = 0.11). There was a higher incidence of satisfactory separation from parents in the dexmedetomidine group (RR = 1.40; 95% CI 1.13–1.74; *p* = 0.002). Dexmedetomidine was also associated with a reduction in the incidence of emergence agitation (RR = 0.35; 95% CI 0.14–0.88; *p* = 0.02). Heart rate and mean arterial pressure were marginally lower in the dexmedetomidine group but without clinical repercussions.

**Conclusion:**

Compared with oral midazolam, intranasal dexmedetomidine demonstrated better separation from parents and lower incidence of emergence agitation in pediatric premedication, without a difference in satisfactory induction. Intranasal dexmedetomidine may be a safe and effective alternative to oral midazolam for premedication in pediatric patients.

## Introduction

The anxiety and stress in pediatric patients undergoing surgical procedures or imaging exams is one of the major challenges for anesthesiologists. Children might be uncooperative or show physical resistance, which may make separation from parents, mask application, and anesthesia induction difficult. In fact, it has been reported that up to 60% of children suffer from fear, stress, or anxiety during the perioperative period or imaging procedures.^1^ Different premedicants, doses, and routes of administration have been studied in clinical practice in order to minimize emotional discomfort and therefore improve parental separation and the induction of anesthesia, as well as to decrease the likelihood of developing postoperative negative behavioral changes.^2^^,^^3^

Midazolam, a short-acting benzodiazepine, is the most commonly prescribed sedative premedication in children due to its widely demonstrated sedative and anxiolytic efficacy, rapid onset, and high metabolic clearance.^4^, ^5^, ^6^ However, it has undesirable effects on pediatric patients, such as cognitive impairment, negative postoperative behavioral changes, and insufficient prevention of Emergence Agitation (EA), which make this drug far from an ideal premedication for this population.^7^^,^^8^

Dexmedetomidine, a highly selective α2-adrenoceptor agonist, is also commonly used as premedication due to its different properties, such as sedation, anxiolysis, analgesia, and minimal respiratory depression.^9^ The intranasal administration of dexmedetomidine has emerged as an attempt to increase the compliance of children receiving the premedication.

Previous meta-analyses assessed the efficacy of midazolam and α2-adrenoceptor agonists in pediatric premedication; however, those studies either included multiple routes of administration^10^ or were limited by the small number of patients and trials.^11^ Herein, we also include recently published trials, perform a Trial-Sequential Analysis (TSA), and provide new evidence to comprehensively assess the efficacy and safety of two common and specific routes of administration: intranasal dexmedetomidine and oral midazolam.

## Methods

This systematic review and meta-analysis were conducted and reported based on the Preferred Reporting Items for Systematic Reviews and Meta-analyses (PRISMA) and the Cochrane Handbook for Systematic Reviews of Intervention guidelines.^12^^,^^13^ The predefined protocol of the present study was registered in the International Prospective Register of Systematic Reviews (PROSPERO; identifier CRD42023446844).

### Eligibility criteria

The inclusion criteria were as follows: 1) Randomized Controlled Trials (RCTs); 2) That included pediatric patients (≤ 18 years-old); 3) Comparing intranasal dexmedetomidine with oral midazolam, and 4) Reporting at least one of the outcomes of interest. Exclusion criteria were any non-randomized studies (observational or retrospective cohorts), trial protocols, abstracts without peer-reviewed manuscript publications, and studies presenting patients older than 18 years old. Endpoints of interest included the efficacy outcomes – satisfactory separation from parents and satisfactory induction or mask acceptance – and safety outcomes: the incidence of EA, Heart Rate (HR), and Mean Arterial Pressure (MAP).

### Search strategy

Eligible trials were systematically searched using the MEDLINE, Embase, and Cochrane electronic databases. We also searched Google Scholar for non-indexed relevant publications. The final search had no restrictions on language, publication year, country of origin, or journal. The complete literature search strategy is listed in Table S1.

### Study selection

Two independent reviewers (EM and TN) selected eligible studies based on the inclusion and exclusion criteria, and a cross-section was performed using the Rayyan software.^14^ After removing duplicates, all results were pooled and selected based on their titles or abstracts. Finally, the remaining articles were read in full to assess eligibility. Any disagreement between the two reviewers was resolved through a discussion with a third reviewer (CG).

### Data extraction

After study selection, the final RCTs included underwent data extraction to summarize the following variables: author, publication year, type of surgery or procedure, dose of intranasal dexmedetomidine and oral midazolam, number and age range of participants, the result of outcomes of interest, and the scale used for outcomes measurements. When continuous data were reported as a median and interquartile range, the values were converted to mean and standard deviation using the Wan et al method.^15^ The values reported in the graphs were extracted using PlotDigitalizer (https://plotdigitizer.com/app).

### Risk of bias assessment

Two reviewers (EM and TN) independently evaluated the risk of bias using the revised Cochrane risk of bias tool for randomized trial 2 (RoB 2),^16^ which consists of five categories: 1) Bias arising from the randomization process; 2) Bias due to deviations from intended interventions; 3) Bias caused by missing outcome data; 4) Bias in the measurement of the outcome; and 5) Bias in the selection of the reported result. Risk of bias was classified as “low risk”, “some concerns”, or “high risk”. The Robvis tool was used to create the final figure.^17^ Any disagreements were resolved by consensus through discussion among all authors.

### Certainty of evidence

The Grading of Recommendations Assessment, Development, and Evaluation (GRADE)^18^ system was used to assess the evidence's certainty level. This system comprises five domains: risk of bias, inconsistency, indirectness, imprecision, and publication bias. The overall quality was classified as high, moderate, low, or very low. The quality of all outcomes was assessed by two independent reviewers (EM and TN), and any disagreements were resolved through discussion among all authors.

### Statistical analysis

Review Manager 5.4^19^ was used for data analysis. Risk Ratios (RR) with 95% Confidence Intervals (95% CI) were calculated for dichotomous variables. Mean Differences (MD) with 95% CI were computed for continuous outcomes, such as HR and MAP. A random-effects model was chosen for all outcomes due to anticipated heterogeneity among studies.

Cochran Q test and I^2^ statistics were used to assess for heterogeneity. Heterogeneity among studies was categorized as low (I^2^ = 0–40%), moderate (I^2^ = 30–60%), substantial (I^2^ = 50–90%) or considerable (I^2^ = 75–100%), according to the Cochrane handbook guidelines.^13^ Publication bias was investigated by funnel plot analysis, and Egger's linear regression test^20^ was performed to explore publication bias further when at least ten studies were included in the outcome analysis.

Statistical significance was set at *p* < 0.05. We also performed a sensitivity analysis by omitting each study individually under a random-effects model to identify a possible small study effect or publication bias using the R software (https://www.r-project.org). Furthermore, we performed a TSA to minimize the risk of type I and type II errors and estimate the required information size. A type I error of 0.05 and a type II error of 0.20 (80% power) were allowed. The adjustment of thresholds for the *Z* score was set with the O'Brien-Fleming alpha spending function, and all the outcomes were assessed under a random-effects model (DerSimonian-Laird method). The software TSA version 0.9 beta (http://www.ctu.dk/tsa) was used for the analysis.^21^

## Results

### Study selection and characteristics

**Figure 1:**
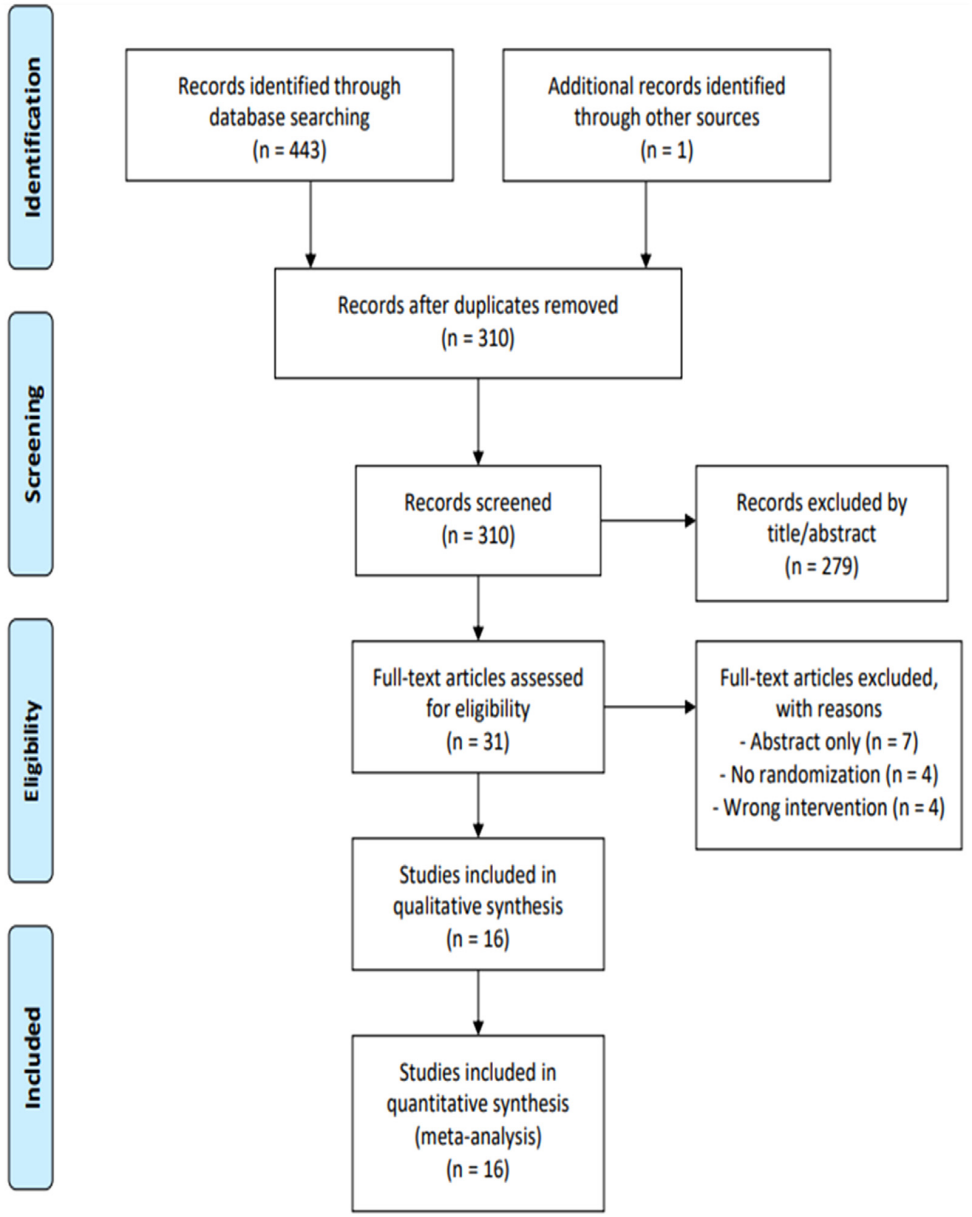
Preferred Reporting Items for Systematic Reviews and Meta-analyses flow diagram of study selection. Four hundred forty-four articles were identified through electronic databases and other sources (non-indexed journals). After removing 310 duplicate studies, 279 articles were excluded based on title or abstract. The full texts of the remaining 31 studies were reviewed, and 15 were excluded from the final selection. Finally, 16 randomized clinical trials were included in the final analysis.

A total of 444 articles were initially identified. Of these, 134 were removed owing to duplication, and 279 were excluded after title and abstract screening. The remaining 31 articles were read in full, and 15 studies were excluded based on the inclusion and exclusion criteria. The final analysis included 16 publications in total (Fig. 1).^22^, ^23^, ^24^, ^25^, ^26^, ^27^, ^28^, ^29^, ^30^, ^31^, ^32^, ^33^, ^34^, ^35^, ^36^, ^37^ The study included 1239 pediatric patients, of whom 643 (51.9%) and 596 (48.1%) were assigned to the intranasal dexmedetomidine and oral midazolam group, respectively.

One study included only children who underwent imaging tests (computed tomography scans),^24^ whereas other studies had pediatric patients who underwent elective surgeries. All studies included a midazolam dose of 0.5 mg.kg^−1^, and the intranasal dexmedetomidine dose ranged from 0.5 to 2 μg.kg^−1^. The characteristics of the RCTs included, and the scales for the outcome measurements are summarized in Table 1.

**Table 1:** Baseline characteristics of included RCTs.

Author, Year	Type of procedure	Patients, n DEX/MID	Age, years (range)	Male, % DEX/MID	DEX dose (μg.kg^−1^)	Scale for parental separation satisfaction	Scale for induction satisfaction	Scale for emergence agitation
Bromfalk, 2021^22^	ENT surgery	30/27	2 ‒ 6	57/63	2	Not measured	10-point scale	Not measured
Cai, 2021^23^	Lower abdominal or perineal surgery	46/37	2 ‒ 6	89/92	2	4-point PSAS	4-point MAS	PAED
Ghai, 2016^24^	CT scan	30/29	1 ‒ 6	30/48	2.5	3-point scale	Not measured	Not measured
Ghali, 2011^25^	Elective adenotonsillectomy	60/60	4 ‒ 12	57/47	1	mYPAS	Not measured	Not measured
Jambure, 2016^26^	Cardiac catheterization	31/30	2 ‒ 10	87/63	2	Not measured	Not measured	Not measured
Kumar, 2017^27^	Abdominal surgery	30/30	2 ‒ 12	50/63	1	6-point scale	6-point scale	4-point scale
Rani, 2017^28^	Elective surgery	32/32	4 ‒ 12	94/94	1	MOAA/S	MOAA/S	Not measured
Sathyamoorthy, 2019^29^	Dental surgery	36/37	5 ‒ 18	67/70	2	UMSS	4-point MAS	Not measured
Savla, 2014^30^	Elective surgery	19/15	1 ‒ 6	95/93	2	Not measured	RSS	Not measured
Segovia, 2014^31^	Elective surgery	52/56	2 ‒ 12	46/57	1	Not measured	mYPAS	mYPAS
Singh, 202^32^	Dental surgery	51/51	4 ‒ 7	47/51	2	4-point PSAS	4-point MAS	Not available
Talon, 2009^33^	Reconstructive Surgery	50/50	1 ‒ 18	46/60	2	Ramsey-like 4-point scale	4-point scale	4-point scale
Wang, 2020^34^	Dental surgery	30/30	3 ‒ 6	53/50	2	4-point PSAS	4-point MAS	PAED
Yadav, 2019^35^	Minor surgery	30/30	1 ‒ 8	50/60	0.5	Not measured	Not measured	Not measured
Yao, 2020^36^	Strabismus surgery	52/50	2 ‒ 6	58/66	2	Not measured	Not measured	PAED
Yuen, 2008^37^	Elective minor surgery	64/32	2 ‒ 12	92/94	0.51	MOAA/S	MOAA/S	Not measured

RCTs, Randomized Clinical Trials; DEX, Intranasal Dexmedetomidine group; MID, Oral Midazolam group; ENT, Ear, Nose and Throat; PSAS, Parental Separation Anxiety Scale; MAS, Mask Acceptance Scale; PAED, Pediatric Anesthesia Emergence Delirium scale; mYPAS, Modified Yale Preoperative Anxiety Scale; UMSS, University of Michigan Sedation Scale; RSS, Ramsay Sedation Scale; MOAA/S, Modified Observer's Alertness/Sedation scale; CT, Computerized Tomography.

### Efficacy outcomes

**Figure 2:**
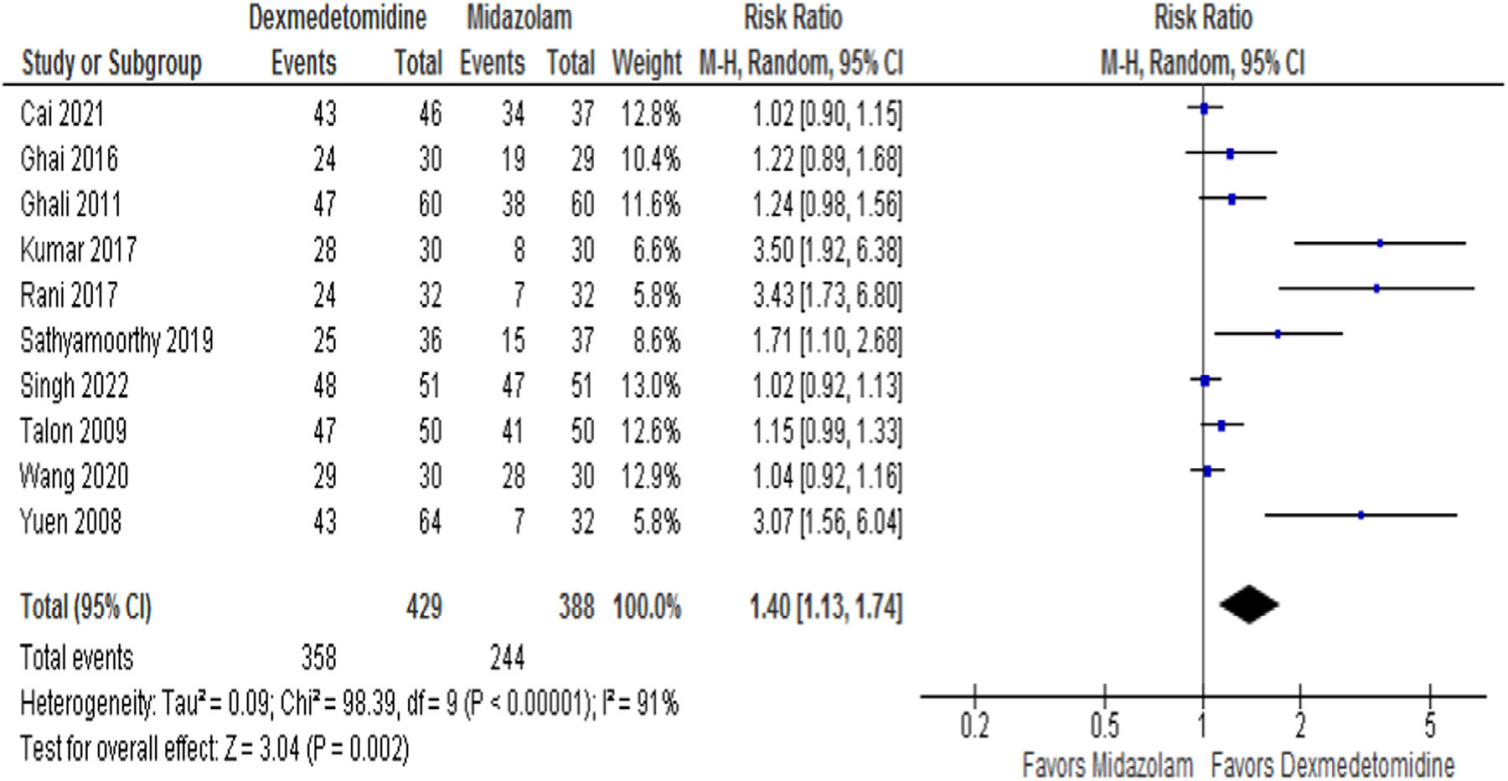
Forest plot for satisfactory separation from parents. Patients in the intranasal dexmedetomidine group showed better parental separation in comparison to patients in the oral midazolam group.

**Figure 3:**
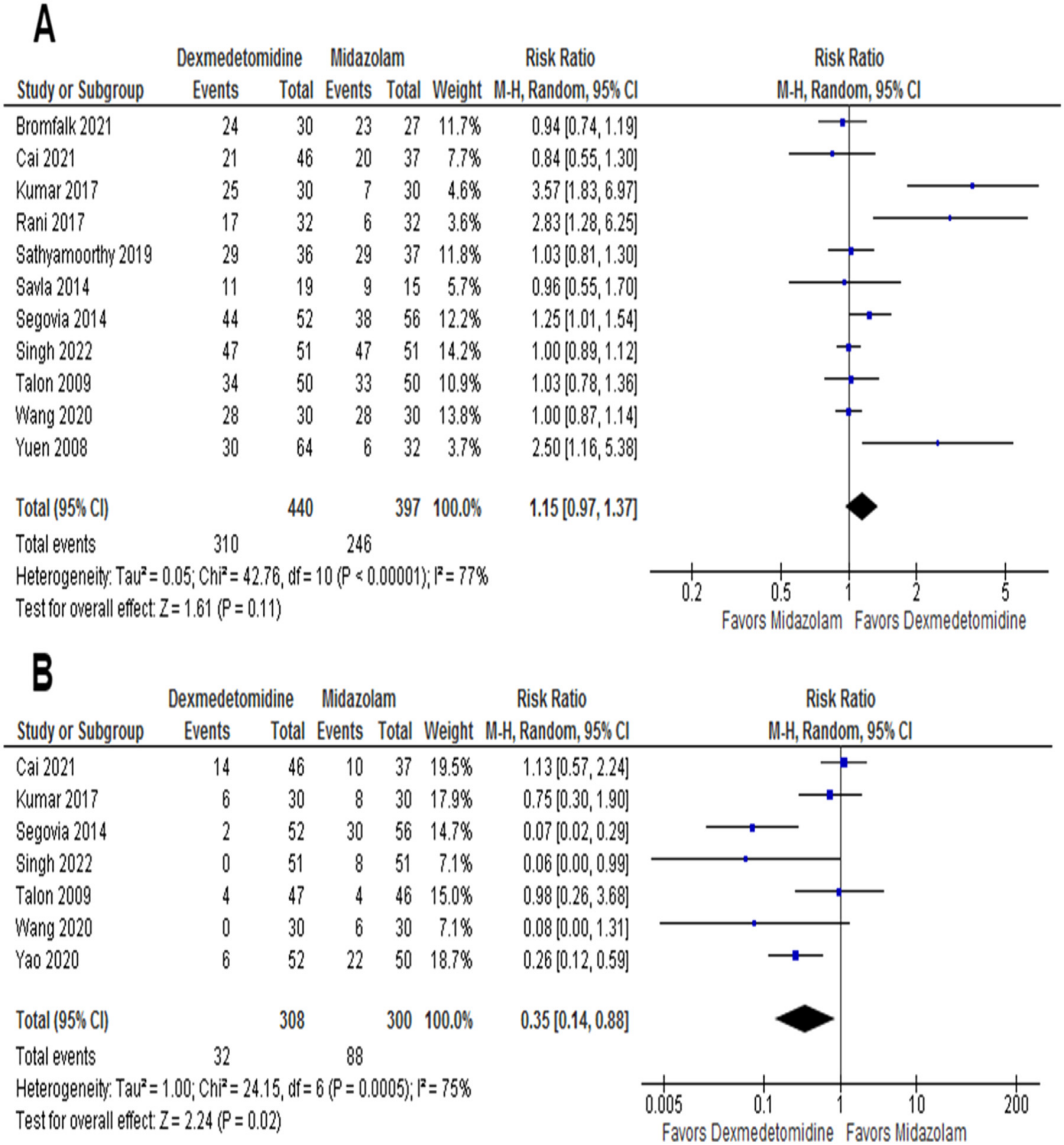
(A) Forest plot for satisfactory induction or mask acceptance. No statistically significant difference was found between both groups. (B) Forest plot for emergence agitation. The intranasal dexmedetomidine group showed less incidence of emergence agitation compared with the oral midazolam group.

The satisfactory separation from parents was assessed by ten RCTs, with 817 patients. Satisfactory parental separation was achieved in 83.5% of the intranasal dexmedetomidine group and 62.9% of the patients in the oral midazolam group. The pooled effect size significantly increased (RR = 1.40; 95% CI 1.13–1.74; *p* = 0.002; I^2^ = 91%) in satisfactory parental separation favoring intranasal dexmedetomidine (Fig. 2). Of the 16 RCTs, 11 reported satisfactions at the time of anesthesia induction or mask acceptance, with 837 patients. The incidence of satisfactory induction or mask acceptance in the intranasal dexmedetomidine and the oral midazolam group was 70.5% and 62%, respectively. However, there was no statistically significant difference in the pooled effect size (RR = 1.15, 95% CI 0.97–1.37; *p* = 0.11; I^2^ = 77%; Fig. 3A).

### Safety outcomes

**Figure 4:**
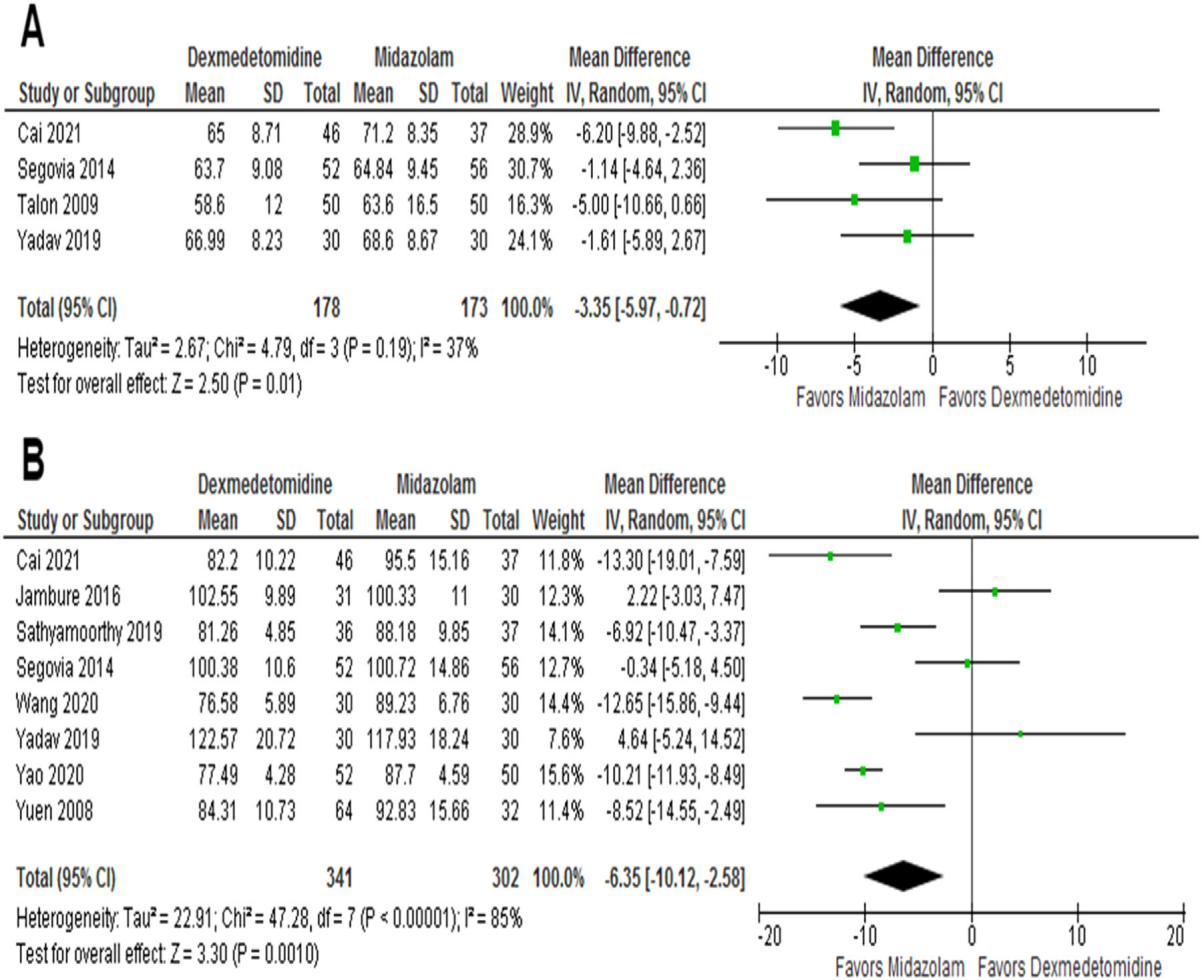
(A) Forest plot for mean arterial pressure. Patients who received intranasal dexmedetomidine showed a greater reduction in MAP compared with the oral midazolam group. (B) Forest plot for heart rate. The intranasal dexmedetomidine group showed a greater reduction in HR in comparison with the patients who received oral midazolam.

The incidence of EA was reported in seven studies, with 608 patients included. EA was significantly reduced in the intranasal dexmedetomidine group (10.3%), as compared with the oral midazolam group (29.3%) (RR = 0.35; 95% CI 0.14–0.88; *p* = 0.02, I^2^ = 75%) (Fig. 3B). A total of four RCTs, with 351 patients, reported the MAP. Intranasal dexmedetomidine was associated with a greater reduction in MAP compared to oral midazolam (MD = -3.35 mmHg; 95% CI -5.97 to -0.72 mmHg; *p* = 0.01, I^2^ = 37%) (Fig. 4A). The HR was reported in eight RCTs with 643 patients. The HR was also lower in the group that received intranasal dexmedetomidine (MD = -6.35 bpm; 95% CI -10.12 to -2.58 bpm; *p* = 0.001, I^2^ = 85%) (Fig. 4B).

### Heterogeneity and sensitivity analysis

Visual inspection of the funnel plot for the outcome of satisfactory separation from parents (Fig. S1) showed considerable heterogeneity, likely from a small study effect carried out by three studies,^27^^,^^28^^,^^37^ and the sensitivity analysis (Fig. S2) confirmed the heterogeneity attributed to these same studies. Egger's test consistently reached statistical significance, suggesting publication bias (*p* < 0.0001). However, despite the high heterogeneity, the pooled effect size remained significant with the exclusion of any individual study. Similarly, qualitative assessment of the funnel plot for satisfactory induction or mask acceptance (Fig. S3) suggests a small study effect, and Egger's test for publication bias was also significant (*p* = 0.038). Still, the sensitivity analysis showed that excluding any study would not create the statistical significance of the pooled effect size (Fig. S4).

### Risk of bias

The risk of bias summary and the overall plot of the risk of bias are shown in Figure S5. The risk of bias was classified as “some concerns” in three studies^26^, ^28^, ^32^ and low in the remaining 13 studies. All included RCTs randomly assigned the patients to the groups, but in three studies,^26^^,^^32^^,^^37^ the authors did not clearly describe whether the allocation sequence was concealed until all participants were assigned to the intervention. Bias due to deviations from the intended intervention was a concern in two studies^32^, ^33^ due to uncertain caregiver blinding, and bias in the measurement of outcomes was also assigned as “some concerns” in one study^32^ due to unclear outcome measurement. Bias due to missing outcome data was assessed as low in all studies, and bias in the selection of reported results was classified as “some concerns” in three studies.^26^^,^^28^^,^^32^

### TSA

**Figure 5:**
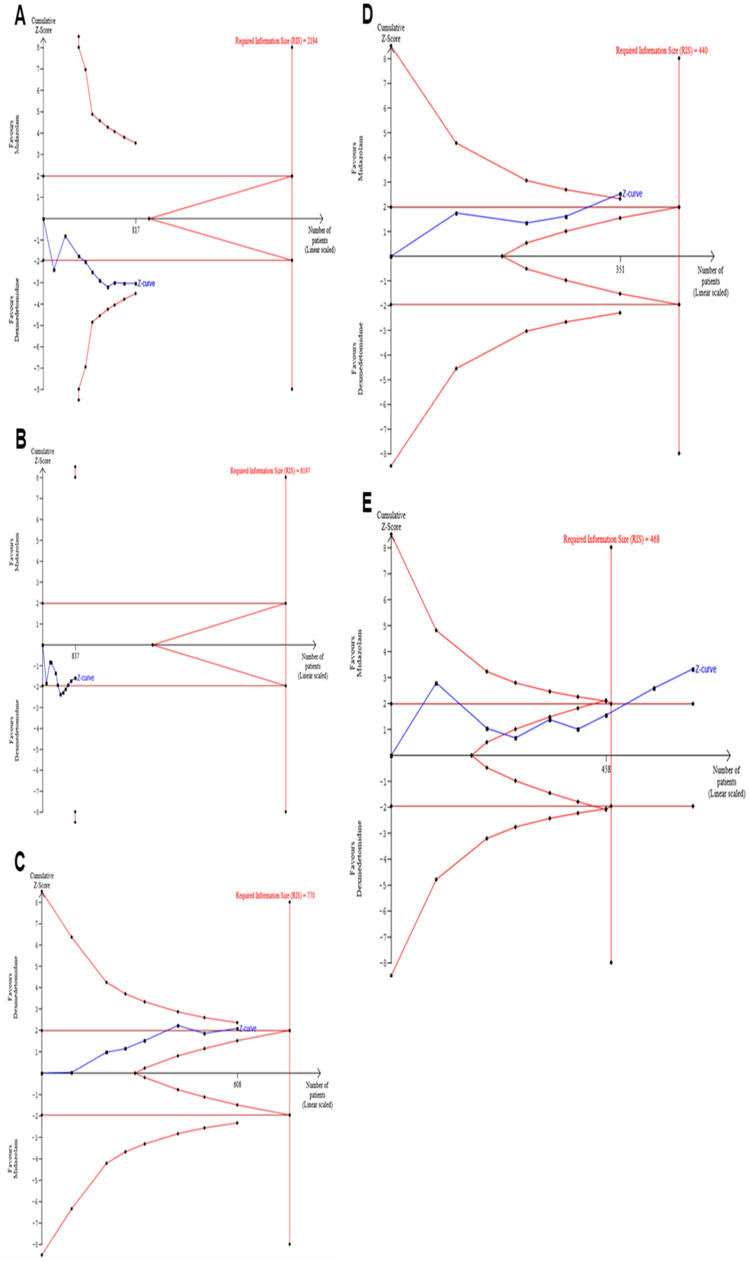
The trial-sequential analysis. for the outcomes analyzed. (A) Satisfactory separation from parents. (B) Satisfactory induction or mask acceptance (three trials^27^, ^30^, ^34^ were ignored in the interim due to too low information use [<1.0%]). (C) Incidence of emergence agitation. (D) Mean arterial pressure. (E) Heart rate. None of the graphs about efficacy outcomes and emergence agitation crossed the trial sequential monitoring boundaries and did not reach the required information size. The monitoring boundaries were crossed in the analysis of mean arterial pressure and heart rate, and the required information size was achieved for heart rate.

The TSA for both safety outcomes and EA did not cross the trial sequential monitoring boundaries or reach the required information sample size (Figs. 5A, 5B, and 5C). In the analysis of satisfactory induction or mask acceptance, three trials^27^, ^30^, ^34^ were ignored in the interim due to too little information use (< 1.0%). However, the monitoring boundaries were crossed in the assessment of MAP and HR, and the required information size was achieved in the analysis of HR (Fig. 5D and 5A).

### Certainty of evidence

Although the results from the risk of bias assessment partially indicate that the quality of the included RCTs was reasonable, the GRADE assessment demonstrated that the overall level of certainty of the evidence in this meta-analysis was low for the efficacy outcomes and for EA, moderate for HR, and high for MAP. This is primarily due to the high heterogeneity among studies, especially for safety outcomes, and the publication bias suggested by the funnel plot analyses. Table S2 shows the overall GRADE summary of findings for each outcome.

## Discussion

In this meta-analysis, intranasal dexmedetomidine as a premedicant in pediatric patients was associated with 1) A higher rate of satisfactory separation from parents, 2) Lower incidence of EA, and 3) A marginally lower HR and MAP in comparison with the use of oral midazolam. There was no difference in satisfactory induction or mask acceptance between groups.

Although preanesthetic medication is often administered in pediatric patients, the optimal drug and ideal route of administration remain controversial. The most commonly used drug for premedication in children is the benzodiazepine midazolam, especially through the oral route. However, oral administration of midazolam may result in low bioavailability, and there may be low acceptability by children due to its poor palatability.^38^^,^^39^ Intranasal administration of dexmedetomidine, a highly selective α2-adrenoceptor agonist, is a relatively simple and noninvasive method to reduce preoperative anxiety and provide sedation to pediatric patients undergoing invasive procedures or imaging scans. The intranasal route is effective due to the bypass of first-pass metabolism and the abundant blood supply at the nasal mucosa. In addition, the administration of intranasal dexmedetomidine is tasteless and odorless, which increases the rates of acceptability by children and has led to increased popularity in clinical practice over the years.^40^

In a meta-analysis published in 2020 by Lang et al,^10^ the authors compared premedication with dexmedetomidine and midazolam through various routes, including intravenous, inhalational, and sublingual. However, no subgroup of the route of administration was performed, and the findings of two of the most commonly used in clinical practice (intranasal dexmedetomidine and oral midazolam) remained unclear, especially concerning the differences in hemodynamic parameters, which were not compared in-depth in their analysis. The authors even attributed the high heterogeneity in all endpoints to the different administration routes. Although their results were similar to ours, the pediatric age group has physiological and pharmacological particularities. It lacks specific research compared to other anesthesia fields,^41^ which is illustrated by the “off-label” use of dexmedetomidine in pediatric anesthesia. An analysis of specific administration methods, such as the one presented herein, must be performed to confirm robust findings, considering these differences and the current lack of evidence about the ideal route of premedication.^42^

Our study was written concomitantly with a recent meta-analysis published by Zhang et al,^11^ comparing intranasal dexmedetomidine with oral midazolam in pediatric patients. Our results support similar findings regarding successful separation from parents, although with a more in-depth report, such as the TSA and five additional trials (455 new patients). In contrast, the prior work did not assess the incidence of EA and the hemodynamic parameters. In addition, in their analysis, anesthesia induction and mask acceptance were assessed separately, resulting in a small sample of trials (3 and 4, respectively). They found better anesthesia induction in the dexmedetomidine group; however, the analysis was under a fixed-effect model, and our study did not support this finding. Furthermore, unlike the authors, we did not include the study by Schmidt et al,^43^ since dexmedetomidine was administered by the sublingual route and, therefore, may have resulted in different findings.

The results of the present meta-analysis suggest that intranasal dexmedetomidine, when administered as premedication in pediatric patients, provides more successful separation from parents than oral midazolam. However, the TSA did not confirm enough information to draw a definitive conclusion. Despite the high heterogeneity found for this outcome, the sensitivity analysis in our study did not change the pooled effect size with the exclusion of any particular study, which indicates that the presented results are consistent and robust to sensitivity analysis.

Dexmedetomidine, unlike other sedatives, acts in the locus coeruleus, thus providing an easy shift from sleep to arousal, particularly with external stimulations such as in anesthesia induction and mask placement.^44^ Therefore, the absence of difference in the outcomes of satisfactory anesthesia induction and mask acceptance between groups, shown in our study, is reassuring. This indicates no significant loss in the clinical efficacy of premedication with dexmedetomidine despite the easier arousal.

Compared to oral midazolam, intranasal dexmedetomidine reduced the incidence of EA. The high variability observed in our study may have been caused by using different metrics to evaluate the occurrence of EA. However, several etiologies, including the type of operation, age, preoperative anxiety level, timing of pre-medication, and pain management, may contribute to the development of EA.^45^ In our study, the premedicants were given before surgery in all the included trials, and most surgeries were minor elective procedures, which may have helped reduce the incidence of EA. Furthermore, as a highly selective α2-adrenoceptor agonist, dexmedetomidine has analgesic effects that might have contributed to the greater reduction in EA found in our meta-analysis since it is well-known that pain plays an important role in the development of EA.^46^

Our study found that intranasal dexmedetomidine premedication showed a greater intraoperative reduction in both HR and MAP compared to oral midazolam, confirmed by TSA. This contrasts with a previous study comparing intranasal dexmedetomidine with intranasal midazolam, which indicates the differences in administration routes of both drugs.^47^ Notably, hypotension and bradycardia are the most commonly reported adverse events associated with dexmedetomidine. These hemodynamic changes are due to the post-synaptic activation of alpha-2 receptors at the locus coeruleus, which lowers the sympathetic outflow, thus leading to a decrease in HR and blood pressure. However, reports of hemodynamic collapses or the need for pharmacological resuscitation remain rare.^48^ Furthermore, dexmedetomidine has been shown to have minimal effects on respiration, which is an important advantage over other agents.^49^ Nonetheless, only one study^26^ stated the occurrence of these adverse events: two episodes of bradycardia and one episode of hypotension in the intranasal dexmedetomidine group, and only one episode of bradycardia in the oral midazolam group. No episodes of bradycardia or hypotension occurred in the other studies; thus, a comparison could not be carried out. Therefore, given that the variations in vital signs were identified in our meta-analysis but that intranasal dexmedetomidine did not appear to be connected with adverse events, it is unlikely that the small difference between groups in HR and MAP has any clinical significance, albeit statistically significant.

This meta-analysis has some limitations. Although only three RCTs^26^, ^28^, ^32^ showed some concerns about the risk of bias assessment, the low and moderate GRADE assessment must be considered when interpreting the results, mainly due to high heterogeneity. Different variables probably carried out this heterogeneity. First of all, there has never been a reliable way to quantify EA or to evaluate anxiety and sedation concerning parental separation, induction, or mask acceptance. Thus, the use of different scales must be taken into consideration. Second, including small, single-center trials with different procedures, doses, ages, and anesthesia protocols may also have caused discrepancies in the results. The absence of patient-level data precluded a more granular assessment of characteristics associated with the relative comparison between groups, including age and dose of anesthetic agent. Due to a lack of reported data, we could not assess additional outcomes, such as the emergence time, time to post-operative care unit discharge, analgesia requirement, and postoperative nausea or shivering. Finally, our results may also be subject to (1) Publication bias, as indicated by the funnel plot analysis and confirmed by Egger's test, and (2) Type I and type II errors, since TSA findings indicated insufficient power and sampling to draw a definitive conclusion for the outcomes of satisfactory separation from parents, satisfactory induction and mask acceptance and incidence of EA. Therefore, further research on these two specific routes of administration is needed, specifically to verify the efficacy and the incidence of EA, preferably with standardized scales.

## Conclusion

In pediatric patients undergoing sedation, premedication with intranasal dexmedetomidine, compared with oral midazolam, improved parental separation satisfaction, lowered the incidence of EA, and was associated with a marginally lower HR and MAP, without a significant difference in satisfactory induction or mask acceptance. Intranasal dexmedetomidine may be considered a safe and effective alternative to oral midazolam for anesthetic premedication in pediatric patients.

## Declaration of competing interest

The authors declare no conflicts of interest.

This research did not receive any specific grant from funding agencies in the public, commercial, or not-for-profit sectors.

## Appendix Supplementary materials

Image, application 1

## Footnotes

Supplementary material associated with this article can be found in the online version at doi:10.1016/j.bjane.2024.844520.
