# Supplementary material for: Comparison of intranasal dexmedetomidine versus oral midazolam for premedication in pediatric patients: an updated meta-analysis with trial-sequential analysis
Source: Braz J Anesthesiol. 2024 May 25;74(5):844520. doi: 10.1016/j.bjane.2024.844520 (PMC11223073; doi:10.1016/j.bjane.2024.844520)

**BJAN-D-24-00053_Supplementary Material**

**Supplementary Table S1** Search strategy and number of results for each database. One additional trial was included by searching Google Scholar for non-indexed publications. The search was performed on September 1, 2023 and was reapplied before the final review to ensure no additional trials were published by then.

| **Search strategy:** | (dexmedetomidine OR "precedex" OR "adrenergic alpha 2 agonist" OR "adrenergic alpha 2 agonists" OR "adrenergic alpha 2 receptor agonist" OR "adrenergic alpha 2 receptor agonists" OR "alpha 2 adrenoceptor agonist" OR "alpha 2 adrenoceptor agonists" OR "alpha 2 receptor agonist" OR "alpha 2 receptor agonists") AND (intranasal) AND (midazolam OR versed OR dormium) AND (child OR children OR infant OR infants OR pediatric OR pediatrics OR paediatric OR paediatrics) |
| --- | --- |
| **Database** | **Number of results** |
| MEDLINE | 85 |
| Embase | 235 |
| Cochrane | 123 |

**Supplementary Table S2** Grading of Recommendations Assessment, Development, and Evaluation (GRADE) assessment of the level of certainty of the evidence.

| **Outcomes** | **№ of participants**  **(studies)** | **Certainty of the evidence**  **(GRADE)** | **Relative effect**  **(95% CI)** | **Anticipated absolute effects** | |
| --- | --- | --- | --- | --- | --- |
|  |  |  |  | **Risk with**  **oral midazolam** | **Risk difference with intranasal dexmedetomidine** |
| Satisfactory induction or mask acceptance | 837  (11 RCTs) | ⨁ ⨁ ◯ ◯  Low ^a,b^ | **RR 0.87**  (0.73 to 1.03) | 705 per 1.000 | **92 fewer per 1.000**  (190 fewer to 21 more) |
| Satisfactory separation from parents | 817  (10 RCTs) | ⨁ ⨁ ◯ ◯  Low ^a,b^ | **RR 0.71**  (0.57 to 0.89) | 834 per 1.000 | **242 fewer per 1.000**  (359 fewer to 92 fewer) |
| Emergence agitation | 608  (7 RCTs) | ⨁ ⨁ ◯ ◯  Low ^a,c^ | **RR 0.35**  (0.14 to 0.88) | 293 per 1.000 | **191 fewer per 1.000**  (252 fewer to 35 fewer) |
| Heart Rate at 30 min | 643  (8 RCTs) | ⨁ ⨁ ⨁ ◯  Moderate ^a^ | - | The mean heart Rate at 30 min was **96.5** BPM | MD **6.35 BPM lower**  (10.12 lower to 2.58 lower) |
| MAP | 351  (4 RCTs) | ⨁ ⨁ ⨁ ⨁  High | - | The mean MAP was  **67.06** mmHg | MD **3.35 mmHg lower**  (5.97 lower to 0.72 lower) |

* The risk in the intervention group (and its 95% Confidence Interval) is based on the assumed risk in the comparison group and the relative effect of the intervention (and its 95% CI).

MAP, Mean Arterial Pressure; CI, Confidence Interval; MD, Mean Difference; RR, Risk Ratio.

**GRADE Working Group grades of evidence**

High certainty: We are very confident that the true effect lies close to that of the estimate of the effect.

Moderate certainty: We are moderately confident in the effect estimate: the true effect is likely to be close to the estimate of the effect, but there is a possibility that it is substantially different.

Low certainty: Our confidence in the effect estimate is limited: the true effect may be substantially different from the estimate of the effect.

Very low certainty: We have very little confidence in the effect estimate: the true effect is likely to be substantially different from the estimate of effect.

**Explanations**

^a^ High heterogeneity (I^2^ > 50%). Downgraded by one level for inconsistency.

^b^ Funnel-plot analysis possibly indicates publication bias. Downgraded by one level for publication bias.

^c^ Outcome significantly carried out by study with high risk of bias. Downgraded by one level for risk of bias.

**Figure Supplementary 1** The funnel plot analysis for the outcome of satisfactory parental separation. RR, Risk Ratio; SE, Standard Deviation.


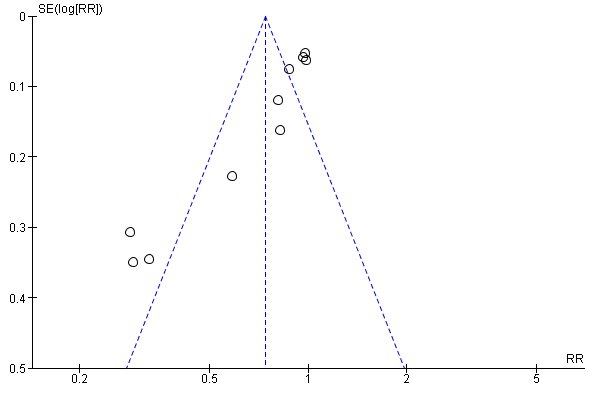


**Figure Supplementary 2** The sensitivity analysis for the outcome of satisfactory parental separation. RR, Risk Ratio; CI, Confidence Interval.


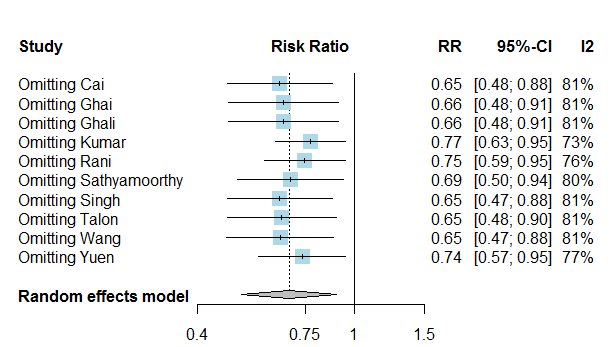


**Figure Supplementary 3** The funnel plot analysis for the outcome of satisfactory induction or mask acceptance. RR, Risk Ratio; SE, Standard Error.


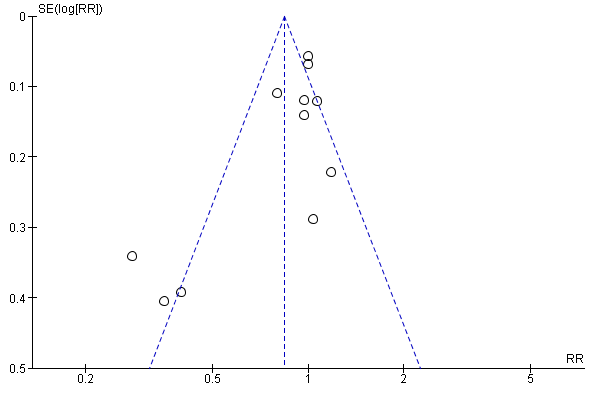


**Figure Supplementary 4** The sensitivity analysis for the outcome of satisfactory induction or mask acceptance. RR, Risk Ratio; CI, Confidence Interval.


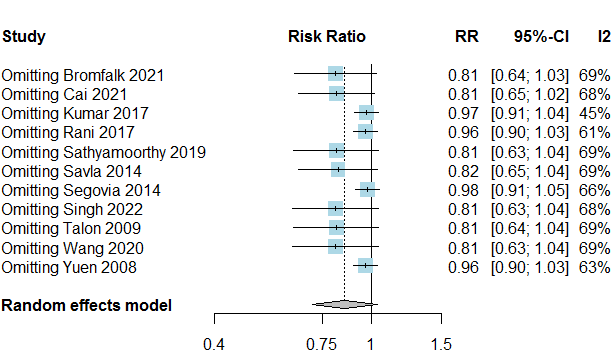


**Figure Supplementary 5** The risk of bias summary and the overall summary plot of the risk of bias.


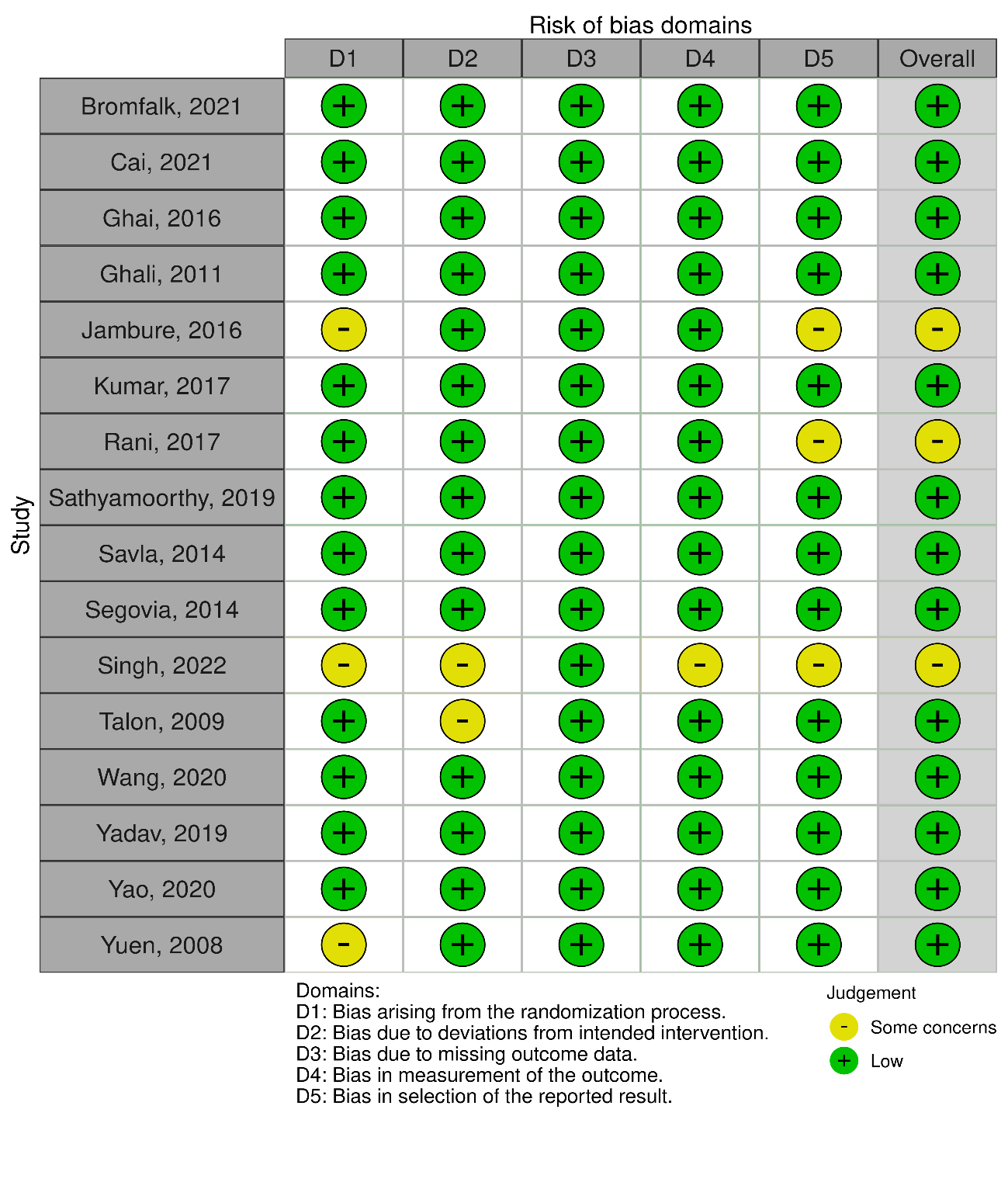

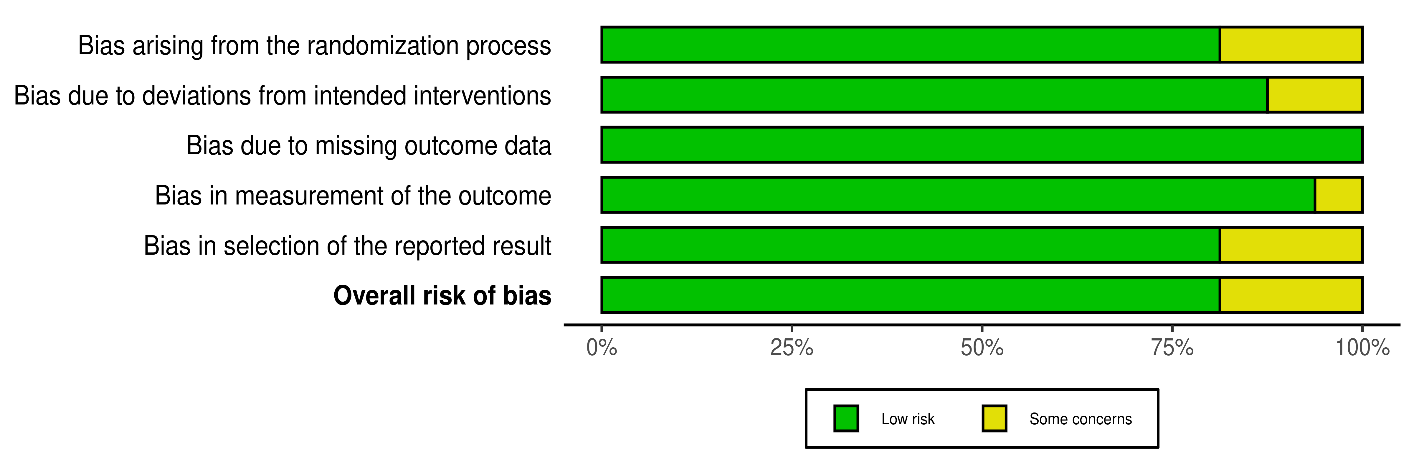

Supplement: Supplementary file 1 [file mmc1.docx]
